# Hemoglobin-to-Red-Cell Distribution Width Ratio Is a Novel Predictor of Long-Term Patient Outcomes After Percutaneous Coronary Intervention: A Retrospective Cohort Study

**DOI:** 10.3389/fcvm.2022.726025

**Published:** 2022-02-16

**Authors:** Wen-Juan Xiu, Ying-Ying Zheng, Ting-Ting Wu, Xian-Geng Hou, Yi Yang, Yi-Tong Ma, Xiang Xie

**Affiliations:** ^1^Department of Cardiology, First Affiliated Hospital of Xinjiang Medical University, Urumqi, China; ^2^Key Laboratory of Cardiac Injury and Repair of Henan Province, Department of Cardiology, First Affiliated Hospital of Zhengzhou University, Zhengzhou, China

**Keywords:** hemoglobin-to-red-cell distribution width ratio, coronary atherosclerotic heart disease, percutaneous coronary intervention, mortality, all-cause mortality

## Abstract

**Background:**

The hemoglobin level and red cell distribution width (RDW) have been linked to the prognosis of coronary atherosclerotic heart disease (CAHD). However, the relationship between the ratio of hemoglobin to the RDW (HRR) and clinical outcomes after percutaneous coronary intervention (PCI) is not known. Here, we explored the impact of the HRR on clinical outcomes after PCI.

**Methods:**

In our study, we selected 6,046 CAHD patients with PCI hospitalized in the First Affiliated Hospital of Xinjiang Medical University from 2008 to 2016. The patients were grouped according to their HRR ratio: group A (HRR < 10.25, *n* = 2,344) and group B (HRR ≥ 10.25, *n* = 3,702). The difference in clinical outcomes between the two groups was compared. Patients were followed up for 35.9 ± 22.6 months.

**Results:**

Three hundred nine patients died during follow-up. These included 166 patients (7.1%) in the HRR < 10.25 group and 143 patients (3.9%) in the HRR ≥ 10.25 group (*P* < 0.001). The incidences of cardiogenic death (5.7 vs. 3.2%) and major cardiovascular adverse events (16.5 vs. 12.9%) also differed significantly between the groups (both *Ps* < 0.001). Analysis using the multivariate Cox proportional hazard model found a significant association between a decreased HRR and post-PCI mortality (all-cause death, adjusted HR: 1.479, 95% CI: 1.156–1.893, *p* = 0.002; cardiac death, adjusted HR: 1.470, 95% CI: 1.116–1.936, *p* = 0.006).

**Conclusion:**

The HRR is predictive of post-PCI mortality among CAHD patients.

## Introduction

Coronary atherosclerotic heart disease (CAHD) is jeopardizing human health with increasing morbidity and mortality rates (1, 2). Multiple risk factors, including gender, age, diabetes, hypertension, smoking, and alcohol consumption, affect the prognosis of CAHD patients (3). Red blood cell (RBC) size variations can be quantified by measuring the RDW (4). The index is calculated automatically by dividing the standard deviation (SD) of the red blood cell volume by the mean red blood cell volume (MCV). Then multiply the result of this equation by 100 and express the result as a percentage (%) (5). The most vital application of RDW is to assess diverse kinds of anemia in the clinic. RDW would rise as well in cases of blood transfusion, chronic liver disease (6), autoimmune disorders (7), and tumor (8). RDW can be used not only as a diagnostic tool, but also as a risk and prognostic factor (4). The RDW has been shown to be predictive of adverse outcomes after acute myocardial infarction (MI) as well as in among patients with stable coronary heart disease (CHD) and heart failure (HF) (9–12).

Tonelli et al. (12) reported a randomized controlled trial that included CHD patients free of HF. The results showed that there was a graded independent relationship between RDW and death (*P* = 0.001 or trend). Compared with those in the lowest quartile, RDW participants in the highest quartile had an adjusted risk ratio of death of 1.78 (95% CI: 1.28–2.47). Osadnik et al. (13) retrospectively analyzed the clinical data of 2,550 CAD patients treated with PCI during 2.5 years of follow-up and found that, after adjusting for risk factors [age, gender, HF, atrial fibrillation, hypertension, previous MI, PCI, coronary artery bypass graft surgery (CABG), and sudden cardiac death (SCD), peripheral vascular disease (PVD), previous stroke, diabetes, lipid abnormalities, fatness, chronic obstructive pulmonary disease (COPD), chronic kidney disease (CKD), smoking, New York Heart Association (NYHA) and CCS class, heart rate, blood pressure, ejection fraction, quantity and kinds of stent implantation, quantity of PCI vessels, hemoglobin, and MCV], higher RDW corresponded to a higher burden of comorbidities and higher mortality.

Like RDW, hemoglobin (Hb) levels also affect CAD prognosis. McKechnie et al. (14) prospectively analyzed 48,851 consecutive patients with PCIs, and data for anemia in 6,471 men (21.7%) and 4,659 women (30.4%) were included. Compared with non-anemic patients, those with anemia had increased risks of postoperative MI and in-hospital mortality as well as higher comprehensive endpoints of major cardiovascular events (MACEs, including MI, cerebrovascular events, and death). This showed that pre-procedural anemia is related to poor prognosis among CAD patients hospitalized after PCI. According to the study by Reinecke et al. (15), 30 patients in the lowest hemoglobin quintile (Hb ≤12.9 g/dl) died at 2 years, corresponding to a mortality rate of 22.2%. In addition, 30 of 62 (48%) all-cause deaths and 19 of 44 (43%) cardiac deaths were in the lowest hemoglobin quintile. Sun et al. (16) retrospectively analyzed 362 esophageal cancer patients and found that the HB/RDW ratio correlated significantly with survival outcomes. The mortality of patients with low HB/RDW was 1.416-fold higher in the follow-up period than that of patients with high HB/RDW. As we all know, HB and RDW are affected by various non-cancer-related conditions, and theoretically reflect broad health information, such as nutritional status, inflammatory status, oxidative stress, and immune function. These underlying pathobiological processes have been shown to predict the outcome of cardiovascular disease (11, 17).

An investigation by Tham et al. (18) showed that the use of Hb/RDW for determining the prognosis of patients with head and neck cancer significantly affected event-free survival. This is consistent with the results of Sun et al., who demonstrated the influence of Hb and RDW on the prognosis of CAD. We hypothesized that the ratio of hemoglobin to RDW (HRR) may affect the prognosis of CAD patients and investigated the relationship between the HRR and the prognosis of CAD patients after PCI.

## Methods

### Study Design and Population

**Figure 1:**
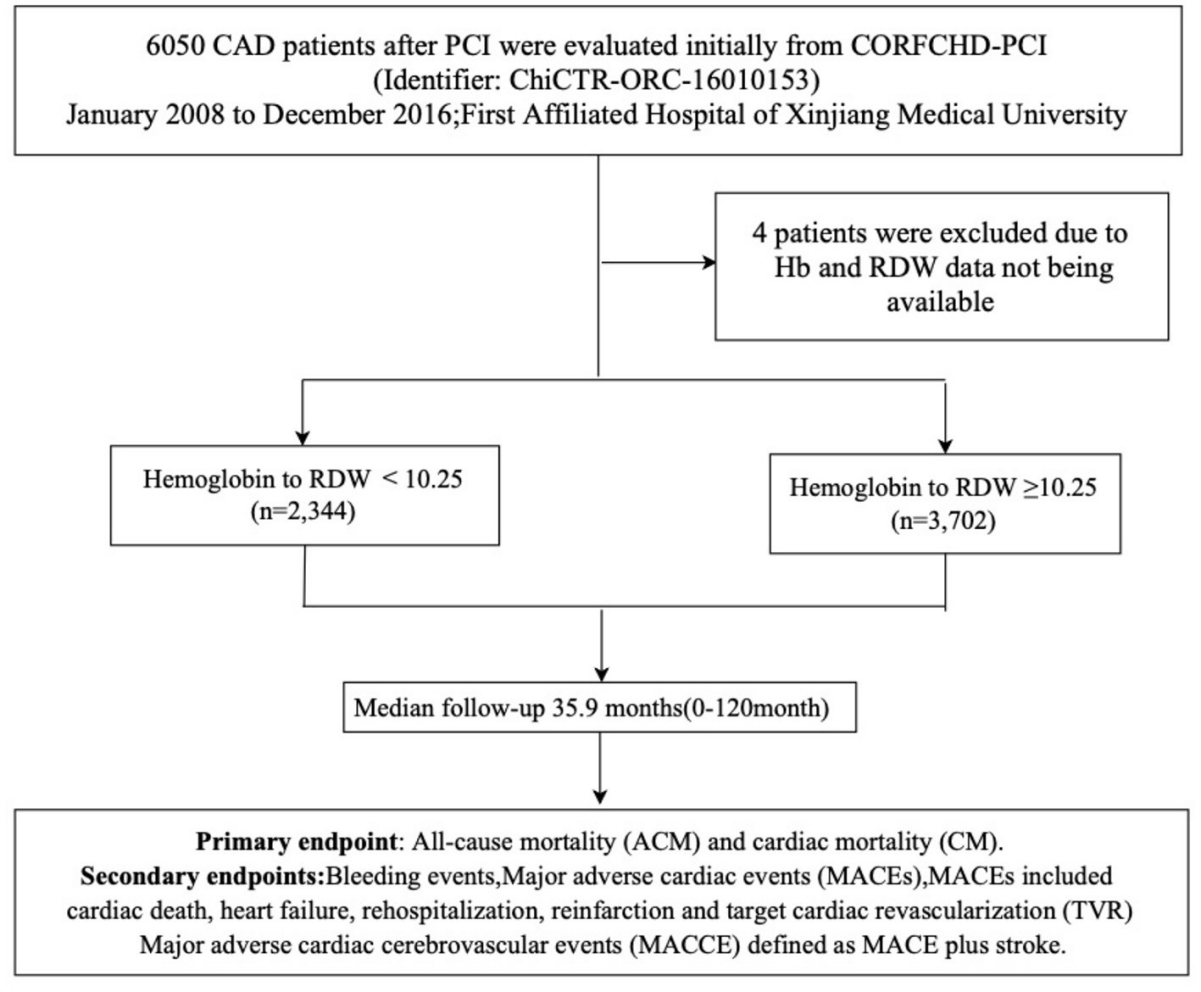
The flowchart of participants inclusion.

CAD patients who had received PCI were evaluated initially from the CORFCHD-PCI study (identifier: ChiCTR-ORC-16010153). The CORFCHD-PCI cohort is a large single-center retrospective cohort comprising 6,050 CAD patients who had undergone PCI and who were from the Clinical Outcomes and Risk Factors of Patients with CHD after PCI at the First Affiliated Hospital of Xinjiang Medical University from January 2008 to December 2016 (19). The inclusion criteria were patients with acute ST-segment elevation myocardial infarction (STEMI), non-ST-segment elevation acute coronary syndrome (NSTE-ACS), and stable angina with coronary angiography and at least one implanted stent. The exclusion criteria were malignant tumors, infectious diseases, hematological diseases, liver diseases, as well as severe heart failure, congenital, rheumatic, or valvular heart disease, and severe kidney or liver dysfunction. To further clarify the influence of the HRR on clinical results of patients with CHD after PCI, an overall 6,050 patients were assessed at first. Owing to inaccessible Hb and RDW data, we excluded 4 patients. The remaining 6,046 CAD patients who successfully received PCI were selected and divided into the HRR <10.25 (*n* = 2,344) and HRR ≥ 10.25 (*n* = 3,702) groups. As this was a retrospective cohort study, the need for patient informed consent was waived. The research protocol was approved by the ethics committee of the First Affiliated Hospital of Xinjiang Medical University and conformed to the guidelines of the Declaration of Helsinki of the World Medical Association. The flowchart of the inclusion and exclusion criteria used in the selection of participants is shown in Figure 1.

### Clinical and Demographic Data

The patients' clinical data, including sex, age, blood urea nitrogen (BUN), creatinine (Cr), uric acid (UA), triglycerides (TGs), total cholesterol (TC), glucose (GLU), high-density lipoprotein cholesterol (HDL-C), low-density lipoprotein cholesterol (LDL-C), red blood cell specific volume (HCT), RBCs, RDW, drinking and smoking status, diabetes, hypertension, CAD family history, and medication history, were recorded and collected. The HRR was defined as Hb (g/L) divided by RDW (%), both from the same blood specimen. Hypertension was defined according to the American Heart Association recommendations (20) as a history of hypertension, the use of antihypertensive medication, or having had three resting blood pressure measurements ≥140/90 mmHg on at least two separate occasions. The diabetes diagnosis was proposed by the WHO in 1999: symptoms of diabetes, intravenous plasma glucose ≥11.1 mmol/L (200 mg/dl) at any time, fasting venous plasma glucose ≥7.0 mmol/L (126 mg/dl), and 2-h intravenous plasma glucose ≥11.1 mmol/L in an OGTT (75 g anhydrous glucose). Regarding the above criteria, diabetes could be diagnosed as long as one of the three was met, any one of the three items was measured again on the following day, and the result of the measurement also met the criteria (21). Hyperlipidemia usually refers to elevated serum cholesterol and/or TG levels, as well as various dyslipidemias, including low HDL-c (22). The assessment of smoking and drinking has been described in detail in the article by Xie et al. (23).

### Endpoints

All-cause mortality (ACM) and cardiac mortality (CM) during follow-up (10 years) were the primary endpoints. The secondary endpoints were MACEs, cerebrovascular events (MACCEs), and bleeding events. MACEs included cardiac death, HF, rehospitalization, reinfarction, and target vessel revascularization (TVR). MACCEs were defined as MACEs plus stroke. Rehospitalization refers to re-admission for cardiovascular disease. All-cause death included both cardiac death and non-cardiac death. The death was classified in all-cause death if with a clear non-cardiac cause of death; otherwise, death was considered to be cardiac death. The criteria for determining bleeding events were derived from the Bleeding Academic Research Consortium standard (24). Reinfarction refers to acute MI that occurs within 28 days of an event or recurrent MI. When ST elevation is ≥0.1 mV or new pathological Q waves appear again in at least two consecutive leads, especially when ischemic symptoms persist for 20 min or more (25). Heart failure with reduced ejection fraction (HFrEF) ≤40 also referred to as systolic HF. Randomized controlled trials have mainly enrolled patients with HFrEF (26). In our study, HF also refers to systolic HF. The definitions of stroke and TVR have been explained in detail in the study by Park et al. (27).

### Follow-Up

In this study, the investigators followed up with patients through telephone contact or an outpatient service. There was tracking survey for each case for 2–10 years, specifically at 1, 3, and 6 months and 1, 3, and 5 years after PCI. The mean tracking survey period registered 35.9 months.

### HRR Detection

Our study used the cutoff finder developed by Budczies et al. (28) to determine the optimal cutoff point for the continuous variable HRR. In our study, cut-off values of ROC curves for HRR were 10.25. According to cutoff value of HRR. The patients were divided into two groups: CAHD patients who successfully received PCI were selected and divided into the HRR < 10.25 (*n* = 2,344) and HRR ≥ 10.25 (*n* = 3,702) groups.

### Statistical Analysis

Continuous variables are presented as the means ± standard deviations and were contrasted by Student's *t*-test; categorical variables are presented as numbers and percentages and were contrasted by the χ-square test. The cumulative incidence of long-term follow-up results was analyzed using Kaplan-Meier analysis. The difference between the groups was compared with the logarithmic test. Multivariable analysis was performed to assess the prognostic value of the HRR and adverse outcomes after adjusting for confounders, such as sex, age, smoking, alcohol drinking, Diabetes, Hypertension, BUN, Cr, and UA, calculating the hazard ratios (HRs) and 95% confidence intervals (CIs). *P* < 0.05 was considered statistically significant. All data were analyzed using SPSS version 22.0.

## Results

### Baseline Characteristics of Patients

Patients were grouped according to their median HRR: The HRR < 10.25 (*n* = 2,344) group and the HRR ≥ 10.25 (*n* = 3,702) group. Patients showed significant differences in terms of gender, age, smoking, drinking, diabetes, hypertension, lymphocyte percentage, RBC specific volume (HCT), RDW, creatinine (Cr), UA, and BUN (all *p* < 0.05). Other baseline data did not differ significantly (*p* > 0.05) (Table 1).

**Table 1:** Characteristics of participants of the two groups.

**Variables**	**Hemoglobin/RDW radio**			
	**<10.25**	**≥10.25**	**Chi-square or** ***t***	***p*** **-value**
Sex, female, *n* (%)	1,037 (44.2)	514 (13.9)	693.452	**<0.001**
Smoking, *n* (%)	648 (27.6)	1,771 (47.8)	243.849	**<0.001**
Age, years	63.15 ± 10.13	57.17 ± 10.62	21.720	**0.001**
Alcohol drinking, *n* (%)	453 (19.3)	1,313 (35.5)	180.846	**<0.001**
Diabetes, *n* (%)	607 (25.9)	844 (22.8)	7.549	**0.003**
Hypertension, *n* (%)	1,059 (45.2)	1,496 (40.4)	13.375	**<0.001**
BMI, kg/?	25.32 ± 3.84	26.37 ± 3.61	3.549	0.060
Hb, g/L	125.16 ±12.03	148.96 ±14.37	2.576	0.109
HCT, %	0.38 ± 0.38	0.44 ± 0.34	6.966	**0.008**
RDW, %	13.75 ± 1.13	12.88 ± 0.63	380.681	**<0.001**
Ly1, 10^9^/L	2.36 ± 0.63	1.92 ± 0.76	3.498	**<0.001**
SBP, mm Hg	126.83 ± 18.31	127.14 ± 18.91	1.782	0.837
DBP, mm Hg	76.27 ± 11.11	76.33 ± 11.38	0.915	0.749
BUN, mmol/L	5.69 ± 1.87	5.41 ± 1.54	6.169	**<0.001**
UA, mmol/L	311.48 ± 94.24	330.71 ± 86.69	−7.998	**<0.001**
Cr, μmol/L	74.95 ± 23.55	76.51 ± 18.17	−2.852	**<0.001**
GLU, mmol/L	6.67 ± 3.27	6.51 ± 3.04	1.859	**0.013**
TG, mmol/L	1.88 ± 1.28	1.91 ± 1.25	−0.773	0.803
TC, mmol/L	3.99 ± 1.14	3.93 ±1.08	1.895	0.215
HDL-C, mmol/L	1.03± 0.46	1.01 ± 0.49	1.278	0.792
LDL-C, mmol/L	2.47 ± 0.94	2.44 ± 0.89	1.222	0.165
ACEI or ARB, *n* (%)	531 (22.8)	835 (22.7)	0.003	0.954
β- blocker, *n* (%)	915 (39.2)	1,511 (41.0)	1.950	0.163
CCB, *n* (%)	267 (11.4)	4,263 (11.5)	0.003	0.956
Statins, *n* (%)	1,205 (51.9)	2,051 (55.9)	9.478	**0.002**

*ACEI, angiotensin-converting enzyme inhibitor; ACEI, angiotensin-converting enzyme inhibitor; ARB, angiotensin receptor blocker; BMI, body mass index; ALB, albumi; BUN, blood urea nitrogen; CCB, calcium channel blocker; Cr, creatinine; DBP, diastolic blood pressure; GLU, glucose; HDL-C, high- density lipoprotein cholesterol; HCT, Red blood cell specific volume; LDL-C, low-density lipoprotein cholesterol; Ly1, Lymphocyte percentage; RBC, red blood cell; RDW, Red Cell volume Distribution Width; SBP, systolic blood pressure; TC, total cholesterol; TG, triglyceride; UA, uric acid. Bold indicates p value < 0.05*.

### Clinical Outcomes

#### Primary Endpoints

##### All-Cause Mortality

The ACM incidence differed significantly between the HRR < 10.25 group and HRR > 10.25 group [166 (7.1%) vs. 143 (3.9%), respectively, *P* < 0.001] (Table 2).

**Table 2:** Outcomes comparison two groups.

**Outcomes**	**Hemoglobin/RDW radio**			
	** <10.25**	**≥10.25**	**Chi-square**	***p*** **-value**
ACM, *n* (%)	166 (7.1)	143 (3.9)	30.699	**<0.001**
CM, *n* (%)	133 (5.7)	118 (3.2)	22.302	**<0.001**
MACCEs, *n* (%)	386 (16.5)	476 (12.9)	15.298	**<0.001**
MACEs, *n* (%)	350 (14.9)	435 (11.8)	12.857	**<0.001**
Heart failure, *n* (%)	85 (3.6)	96 (2.6)	5.275	**0.022**
Stroke, *n* (%)	39 (1.7)	43 (1.2)	2.707	0.100
Bleeding events, *n* (%)	66 (2.8)	109 (2.9)	0.085	0.771
Rehospitalization, *n* (%)	341 (14.5)	478 (12.9)	3.280	0.070
Reinfarction, *n* (%)	73 (3.1)	121 (3.3)	0.110	0.740
TVR, *n* (%)	112 (4.8)	201 (5.4)	1.240	0.265

*ACM, all-cause mortality; CM, cardiac mortality; MACE, major adverse cardiovascular event; MACCE, major adverse cardiovascular and cerebrovascular event; TVR, target vessel revascularization*.

**Figure 2:**
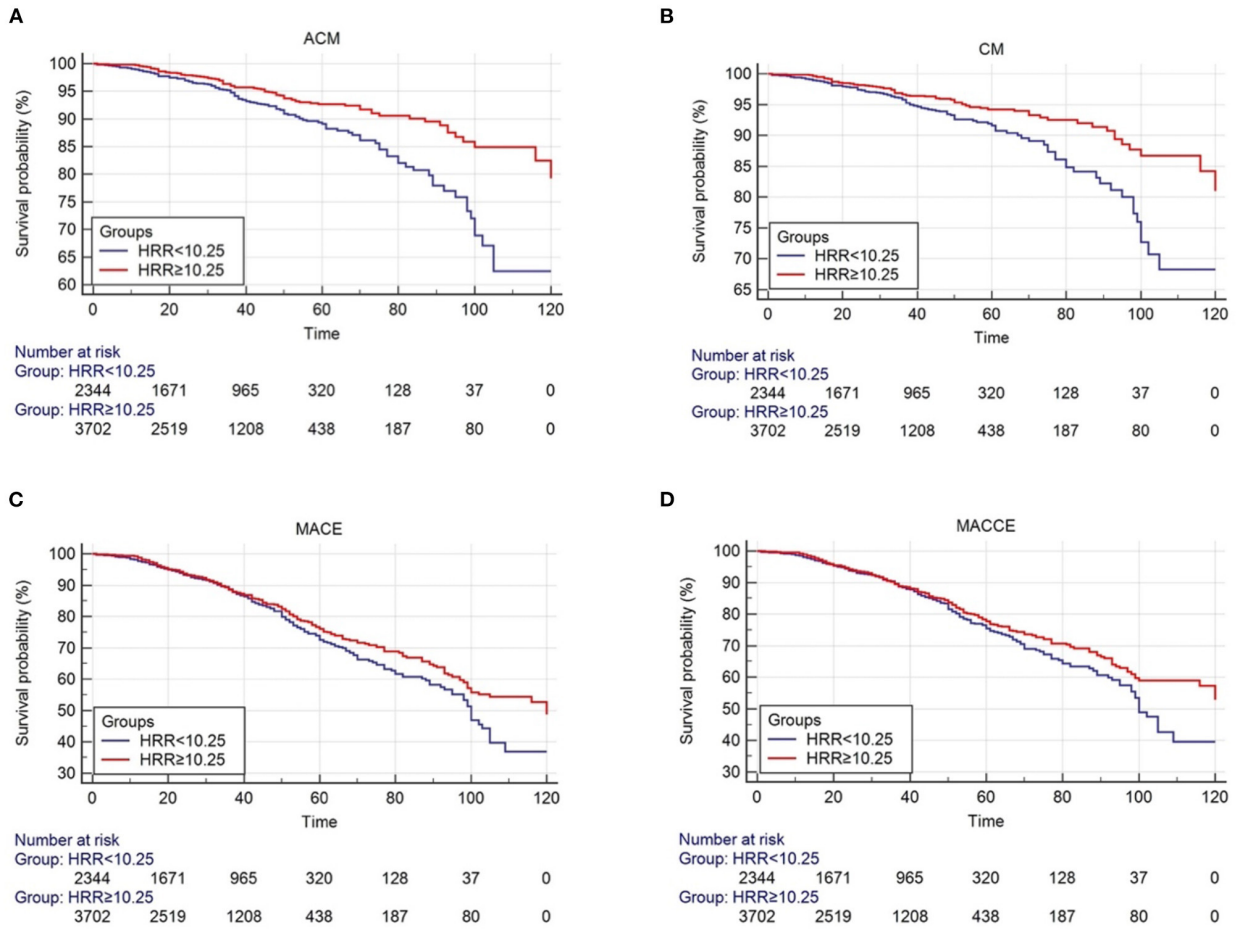
Cumulative Kaplan-Meier estimates of the time to the first adjudicated occurrence of primary endpoint and secondary endpoints. **(A)** ACM; **(B)** CM; **(C)** MACE; **(D)** MACCE.

The survival curve describes the manifestation of the outcome event. Over time, the incidence of endpoint events gradually increases, and the survival rate gradually decreases. The number of risks is used to indicate the number of people exposed to the risk of the outcome at each point in time. The starting point of the study is the total number of people included in the study, 2,344 people in the HRR < 10.25 group and 3,702 people in the HRR ≥ 10.25 group (Figures 2A–D).

As shown by the Kaplan-Meier survival curve and log-rank test, ACM was significantly different between the two groups (*P* < 0.001, Figure 2A). Univariate analysis showed that patients in the HB/RDW radio >10.25 group had a lower risk of all-cause mortality compared with the HB/RDW radio < 10.25 group (HR = 0.590, 95% CI: 0.472–0.739, *P* < 0.001, Table 3). The Cox analysis found the HRR, after adjustment for common risk factors, to be an independent prognostic factor for post-PCI CHD patients in the low HRR group. The incidence of ACM increased 1.47 times (HR = 1.470, 95% CI: 1.116–1.936, *P* = 0.006, Table 4).

**Table 3:** Univariate logistic regression analysis of ACM, cardiac death, MACCEs, and MACEs.

**Characteristics**	**HB/RDW radio** **>** **10.25**	
	**HR (95% CI)**	***p*** **-value**
ACM	0.590 (0.472–0.739)	**<0.001**
CM	0.602 (0.469–0.772)	**<0.001**
MACCE	0.857 (0.749–0.981)	**0.025**
MACE	0.864 (0.750–0.995)	**0.042**
Heart failure	0.796 (0.594–1.066)	0.125
Stroke	0.762 (0.493–1.176)	0.220
Bleeding events	1.124 (0.828–1.528)	0.453
Rehospitalization,	0.975 (0.848–1.120)	0.717
Reinfarction	1.174 (0.878–1.570)	0.280
TVR	1.255 (0.995–1.581)	0.055

*ACM, all-cause mortality; CM, cardiac mortality; MACE, major adverse cardiovascular event; MACCE, major adverse cardiovascular and cerebrovascular event; TVR, target vessel revascularization*.

**Table 4:** Cox proportional hazards analysis of outcomes.

**Characteristics**	**ACM**		**CM**		**MACCEs**		**MACEs**	
	**HR (95% CI)**	***p*** **-value**	**HR (95% CI)**	***p*** **-value**	**HR (95% CI)**	***p*** **-value**	**HR (95% CI)**	***p*** **-value**
Age	1.015 (1.002–1.029)	**0.021**	1.024 (1.012–1.036)	**0.000**	0.997 (0.989–1.004)	0.350	0.998 (0.991–1.005)	0.652
Gender	0.881 (0.628–1.236)	0.462	0.879 (0.648–1.191)	0.405	0.835 (0.684–1.019)	0.076	0.825 (0.684–0.997)	0.046
Smoking	0.881 (0.631–1.230)	0.457	0.989 (0.734–1.332)	0.943	0.899 (0.748–1.080)	0.254	0.849 (0.712–1.011)	0.067
Alcohol drinking	1.007 (0.709–1.431)	0.968	0.955 (0.697–1.309)	0.775	0.920 (0.758–1.115)	0.394	0.930 (0.773–1.119)	0.443
Diabetes	1.126 (0.837–1.515)	0.433	1.030 (0.785–1.350)	0.832	1.199 (1.017–1.414)	0.031	1.197 (1.024–1.401)	0.024
Hypertension	1.139 (0.872–1.487)	0.341	1.212 (0.955–1.539)	0.114	1.366 (1.177–1.585)	**<0.001**	1.354 (1.175–1.560)	**<0.001**
BUN	1.085 (1.007–1.170)	**0.032**	1.062 (0.992–1.137)	0.086	1.061 (1.014–1.110)	**0.010**	1.059 (1.015–1.105)	**0.008**
Cr	1.003 (0.997–1.009)	0.323	1.002 (0.996–1.008)	0.473	0.998 (0.994–1.002)	0.245	0.998 (0.995–1.002)	0.433
UA	1.000 (0.999–1.002)	0.909	1.000 (0.999–1.002)	0.806	1.000 (1.000–1.001)	0.367	1.000 (1.000–1.001)	0.332
HB/RDW radio <10.25	1.470 (1.116–1.936)	**0.006**	1.479 (1.156–1.893)	**0.002**	1.162 (0.994–1.358)	0.060	1.157 (0.997–1.343)	0.054

*BUN, blood urea nitrogen; Cr, creatinine; UA, uric acid*.

##### Cardiac Mortality

CM differed significantly between the HRR < 10.25 group and the HRR > 10.25 group [133 (5.7%) and 118 (3.2%), respectively, *P* < 0.001) (Table 2).

The KM curves and log-rank test demonstrated a significant difference in CM between the HRR < 10.25 group and the HRR > 10.25 (*P* < 0.001, Figure 2B). Univariate analysis showed that patients in the HB/RDW radio >10.25 group had a lower risk of cardiac mortality compared with the HB/RDW radio < 10.25 group (HR = 0.602, 95% CI: 0.469–0.772, *P* < 0.001, Table 3). After risk factor adjustment, the Cox proportional hazards model showed the HRR to be an independent prognostic factor for CAD patients after PCI in the low HRR group. The incidence of CM increased 1.33 times (HR = 1.479, 95% CI: 1.156–1.893, *P* = 0.002, Table 4).

#### Secondary Endpoints

##### MACCEs and MACE

We found that the incidences of MACCEs [386 (16.5%) vs. 476 (12.9%), *P* < 0.001], MACEs [350 (14.9%) vs. 435 (11.8%), *P* < 0.001], and HF [85 (3.6%) vs. 96 (2.6%), *P* = 0.022] differed significantly between the groups. However, the incidences of bleeding events (2.8 vs. 2.9%, *P* = 0.771), stroke (1.7 vs. 1.2%, *P* = 0.100), rehospitalization (14.5 vs. 12.9%, *P* = 0.070), reinfarction (3.1 vs. 3.3%, *P* = 0.740), and TVR (4.8 vs. 5.4%, *P* = 0.265) showed no significant differences (Table 2).

The Kaplan-Meier log-rank tests identified significant differences in the occurrence of MACCEs (*P* = 0.024, Figure 2C) and MACEs (*P* = 0.042, Figure 2D) between the groups. Univariate analysis model also showed that the MACCEs and MACEs were significant differences in the two groups [HR = 0.857 (0.749–0.981), *P* = 0.025 and HR = 0.864 (0.750–0.995), *P* = 0.042, Table 3]. Cox analysis was performed to evaluate the prognostic value of the HRR after adjusting for traditional risk factors, as shown in Table 1. However, MACCEs [HR = 1.162 (0.994–1.358), *P* = 0.060] and MACEs [HR = 1.157 (0.997–1.343), *P* = 0.054] were not significantly different (Table 4). HF [HR = 0.839 (0.607–1.160), *P* = 0.228], stroke [HR = 0.829 (0.515–1.343), *P* = 1.334], bleeding events [HR = 1.058 (0.756–1.480), *P* = 0.742], rehospitalization [HR = 0.975 (0.836–1.136), *P* = 0.742], reinfarction [HR = 1.065 (0.773–1.468), *P* = 0.700] and TVR [HR = 1.115 (0.866–1.436), *P* = 0.400] were not significantly different between the two groups (tables not shown).

## Discussion

This retrospective study of 2,344 (HRR < 10.25) and 3,702 (HRR ≥ 10.25) CAD PCI patients at The First Affiliated Hospital of Xinjiang Medical University from January 2008 to December 2016 provides compelling evidence that the HRR, which is the ratio of Hb to RDW, is a novel and powerful indicator of the clinical outcomes of CAD PCI patients. Low levels of the HRR (HRR < 10.25) increased long-term ACM and CM by 1.470 times and 1.479 times, respectively. In order to reduce the influence of confounding factors on the prognosis of cardiovascular events, we adjusted the comprehensive factors affecting the risk of cardiovascular events to investigate the relationship between the HRR and clinical outcomes. This is the first retrospective cohort study to investigate the influence of the HRR on the prognosis of CAD PCI patients.

The RDW, indicative of variations in RBC size, is known to predict the risk of CVD morbidity and mortality (11, 29, 30). Veeranna et al. (31) selected 8,513 adult participants without CVD from the 1999-2004 National Health and Nutritional Examination Survey (NHANES) to assess the impacts of RDW and high-sensitivity C-reactive protein on future CHD mortality. After adjusting for traditional risk factors, RDW was still an independent predictor. Shah et al. (32) conducted a *post-hoc* analysis of the NHANES 1988-94 cohort, investigating 7,005 non-anemic individuals (aged 30–79 years) to determine the risks of cardiovascular mortality. The analysis identified 27 individuals with RDW > 14.5 (8.2%) and 206 (3.1%) with RDW <14.5 (all *p* < 0.001). Studies have shown that compared with traditional cardiovascular risk factors, RDW is a promising parameter for identifying individuals at risk of cardiovascular disease mortality.

In laboratory examinations, the larger the RDW is, the more the shapes and sizes of RBCs in the sample blood will differ. Exceeding the normal value indicates various anemias, hematopoiesis abnormalities, or congenital RBC abnormalities. A small distribution width indicates that the shape and size of the RBCs in the sample blood are consistent and neat. Although the detection has no obvious specificity, it can still explain the problems in the application scenarios described in the above paper. In short, the widening of the RDW after PCI is the result of uncontrolled inflammation because the outbreak of inflammation is the main factor leading to poor prognosis. Although age, sex, genetic factors, renal function, liver function, inflammation, dyslipidemia, and other factors can lead to an increase in the RDW, the increase is only in the baseline value. The exclusion criteria for this study were patients with malignant tumors, infectious diseases, hematological diseases, liver diseases, as well as severe heart failure, congenital, rheumatic, or valvular heart disease, and severe kidney or liver dysfunction, so the contributions of the above factors to sudden death among the included patients were minimal.

Numerous prospective studies have shown that baseline Hb is a strong prognostic factor for 30-day and 1-year mortality, MACEs, bleeding events, and ischemic events in PCI patients (33, 34). Poludasu et al. (35) conducted a prospective study of 3.2 years and observed an association between baseline Hb and all-cause long-term mortality. Pilgrim et al. (36) found that severe anemia caused a significant increase in the risk of stent thrombosis. Li et al. (37) enrolled a total of 584 among 677 hospitalized patients with angiographic CAD and tracked all patients for 14.3 ± 8.4 months, finding that high Hb levels were independent predictors of MACEs in CAD patients. This shows that a high hemoglobin level independently predicts the prognosis of coronary artery disease.

However, no data are available associating the HRR with CAD risk. Therefore, we explored the relationship between the HRR and prognosis in post-PCI CAD patients. We selected 6,046 CAD patients who had undergone PCI, dividing them according to the HRR median value of 10.25. We found that the HRR is related to the long-term prognosis of CAD patients. Compared with those in the high HRR value group, the incidences of ACM, AM, MACCEs, MACEs, and HF were higher in the low HRR value group. After adjustments for the confounding factors listed in Table 3, we conducted a multivariate Cox analysis. The results showed that the HRR may be an independent predictor of the risk of long-term death in post-PCI CAD patients. Patients with a low HRR have higher ACM and CM. However, it showed limited predictive power in our study [HR = 1.470 (1.116–1.936), *P* = 0.006; HR = 1.479 (1.156–1.893), *P* = 0.002]. Therefore, the result has a high degree of credibility.

However, the mechanism of this correlation is still unclear. Higher RDWs in the normal scope might imply stepped-up RBC disruption or, more often, invalid erythropoiesis (19). A higher RDW also reflects a potential inflammatory state and impaired RBC maturation and is associated with adverse clinical outcomes (38, 39). The RDW is associated with inflammatory markers such as the erythrocyte sedimentation rate and high-sensitivity CRP, interleukin-6, and fibrinogen levels (40). Inflammatory factors can change the homeostasis of RBCs and may inhibit their production and increase the RDW by impairing iron metabolism (41). In patients with higher RDWs, decreased erythrocyte deformability impairs blood flow through the microcirculation (42). In addition, proinflammatory cytokines can lead to iron metabolism disorder and reduce the production of erythropoietin and the response of bone marrow to erythropoietin, resulting in impaired hematopoietic function and increased RDW levels (43–45).

Oxidative stress is another pathophysiological mechanism of RDW in addition to inflammation. An increase in RDW may be accompanied by the loss of RBC deformability and changes in erythrocyte homogeneity, which may damage the transport of RBCs through microvessels, affecting not only the oxygen transport of tissues but also the “antioxidant” function of blood vessels (46). RBCs have a high antioxidant capacity, which may shift toward pro-oxidants when exposed to an environment of high inflammation and oxidative stress, and the change in RBC size is related to oxidative stress, which may be caused by increasing the renewal rate of RBCs (47), thus further aggravating the oxidative burden and accelerating the development of atherosclerosis (46, 48, 49).

There was also a certain correlation between the TC erythrocyte membrane (CEM) level and the RDW (50). Lipid disorder will reduce the fluidity of the erythrocyte membrane, and an increase in CEM levels will lead to a decrease in the cell deformation ability. A higher CEM level will lead to the deterioration of blood flow through the microcirculation (51, 52), affect the lifespan of circulating RBCs, and then lead to greater cell renewal and a higher RDW value (53, 54).

Neutrophil to lymphocyte ratio (NLR) also can reflect the severity of systematic inflammation. NLR is a powerful outcome predictor of vascular disease and has recently been described as a predictor of mortality in patients undergoing percutaneous coronary intervention. The Tamhane study (55) showed that NLR on admission is an independent predictor of hospitalization and 6-month mortality in ACS patients. NLR can perform risk stratification and prognosis assessment for patients with ACS. Previous studies have also shown that NLR is not only a reliable marker of mortality and amputation stratification in patients with acute limb ischemia, but also an independent prognostic factor for the 30-day morbidity of ruptured abdominal aortic aneurysms (rAAA) (56–58).

The hemoglobin level is the main determinant of the oxygen carrying capacity. Impaired oxygen carrying capacity and changes in viscosity or blood flow patterns associated with hemoglobin changes are considered to be the causes of CAD and CAD symptoms. The decrease in the hemoglobin value showed that oxygen delivery to the myocardium downstream of coronary artery stenosis was significantly reduced and that the tissue oxygen supply was limited. Anemia also increases myocardial oxygen demand by increasing the heart rate to maintain adequate systemic oxygen delivery. Anemia patients may show a hypercoagulable state, aggravating the occurrence of ischemic events. Anemia can also lead to ventricular remodeling and hypertrophy and higher oxygen consumption, which is very unfavorable in CHD. Inflammation is very important in CAD, and low hemoglobin levels is a vital indicator of potential inflammatory processes. Similarly, patients with low hemoglobin levels and subclinical CAD may be more prone to symptomatic heart disease (59–61).

At present, it is difficult to determine the factors associated with poor prognosis, including blood lipids, iron metabolism disorder, anemia, insufficient vitamin D3, oxidative stress, inflammation, decreased deformability of the erythrocyte membrane, oxygen carrying capacity, blood viscosity, and changes in blood flow pattern. In CAD patients, the predictive utility of the RDW is that it represents the total of the negative effects of the above reasons on the erythropoiesis function of bone marrow, and the predictive utility of Hb is the sum of the decreased oxygen carrying function of hemoglobin. Therefore, the prognostic value of the HRR reflects the aggravation of these phenomena.

The results of this study have some clinical significance and advantages. Hb and the RDW can be quickly calculated based on routine blood examinations at the time of admission. Based on the HRR results, the patient's health and long-term prognosis can be assessed. Patients with a high BUN and a low HRR should be given more attention, and they may have higher incidences of ACM, MACCEs, and MACEs. This is beneficial to clinicians and lead to better decisions. This study has the following strengths: (1) the sample size was large, improving statistical reliability; (2) There was a low rate of patient loss during the long-term follow-up period. However, the study also has several limitations: (1) we collected only HRR data during the study period, and there were no relevant data in the follow-up results. Therefore, it is impossible to analyze the impact of dynamic changes in these variables; (2) the limitations of the research design require verification by further prospective studies; (3) the cutoff values of Hb/RDW varied widely across previous studies. There has been no clear consensus on the optimal cutoff and the magnitude of association with various outcomes. In addition to unclear pathophysiological mechanisms, these issues may have limited the clinical utility of this novel parameter.

## Conclusion

In conclusion, our findings show the baseline HRR to be an independent predictor of adverse outcomes of post-PCI CHAD patients.

## Data Availability Statement

The original contributions presented in the study are included in the article/supplementary material, further inquiries can be directed to the corresponding author.

## Ethics Statement

The studies involving human participants were reviewed and approved by the Ethics Committee of the First Affiliated Hospital of Xinjiang Medical University. Written informed consent was not required for this study, in accordance with the local legislation and institutional requirements.

## Author Contributions

W-JX and Y-YZ conceptualized the current study objectives, analyzed the data, and wrote the manuscript draft. T-TW and X-GH, and YY collected and organized data. Y-TM and XX had responsibility of the final content. All authors read, approved the final manuscript, and were involved in the conception of the research plan.

## Funding

This research was funded by the National Natural Science Foundation of China (81770235), Xinjiang Science and Technology Aid Project (2019E0278), and Prevention and control of major chronic Non-communicable disease Project (2018YFC1311505).

## Conflict of Interest

The authors declare that the research was conducted in the absence of any commercial or financial relationships that could be construed as a potential conflict of interest.

## Publisher's Note

All claims expressed in this article are solely those of the authors and do not necessarily represent those of their affiliated organizations, or those of the publisher, the editors and the reviewers. Any product that may be evaluated in this article, or claim that may be made by its manufacturer, is not guaranteed or endorsed by the publisher.
